# Supplementary material for: Mortality prediction for ICU patients with mental disorders using large language models ensemble and unstructured medical notes
Source: PLoS One. 2025 Sep 19;20(9):e0332134. doi: 10.1371/journal.pone.0332134 (PMC12448993; doi:10.1371/journal.pone.0332134)
Supplement: Attachment — Submitted filename: Point-by-point response-final-PO.pdf [file pone.0332134.s001.pdf]

Response to the Reviewers on the manuscript entitled: **Mortality Prediction for ICU Patients with Mental Disorders Using Large Language Models Ensemble and Unstructured Medical Notes**

Waleed Nazih, Tamer Abuhmed, Meshal Alharbi, Shaker El-Sappagh

Manuscript ID: PONE-D-24-32699

We thank the reviewers and editor for their valuable comments that significantly helped us to enrich the quality of the manuscript. We've carefully considered the reviewers' comments in our revision. We have tried our best to incorporate the reviewers' and editors' suggestions into the revised version of the manuscript.

To address each reviewer's comments carefully, we would like to respond to them point by point. All changes have been highlighted in the manuscript using the red color.

Best regards,

Authors

Reviewer 1

Comment 1: The Abstract could be more concise and highlight key findings more clearly

Authors response:
Thank you for your feedback. We have revised the abstract, making it more concise and highlighting the main contributions and findings.

Comment 2: Some sentences are overly long and complex, impacting readability
Authors response:
Thank you. We thoroughly revised the entire manuscript and removed overly long and complex sentences

Comment 3: A few grammatical errors and typos need correction
Authors response:
We have revised the manuscript and sent it to a professional academic English editing service. [See Attached Certificate]

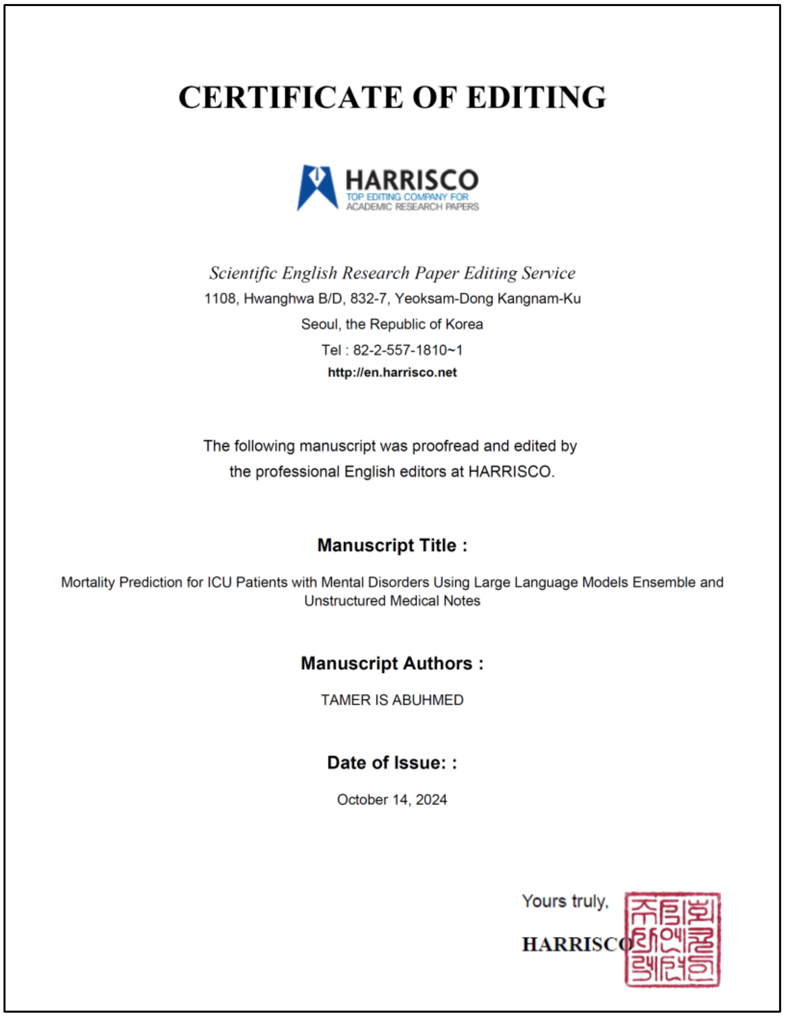

CERTIFICATE OF EDITING

HARRISCO
TOP EDITING COMPANY FOR
ACADEMIC RESEARCH PAPERS

Scientific English Research Paper Editing Service
1108, Hwanghwa B/D, 832-7, Yeoksam-Dong Kangnam-Ku
Seoul, the Republic of Korea
Tel : 82-2-557-1810~1
http://en.harrisco.net

The following manuscript was proofread and edited by
the professional English editors at HARRISCO.

Manuscript Title :

Mortality Prediction for ICU Patients with Mental Disorders Using Large Language Models Ensemble and Unstructured Medical Notes

Manuscript Authors :

TAMER IS ABUHMED

Date of Issue: :

October 14, 2024

Yours truly,

HARRISCO

Comment 4: Figures could be improved for clarity, especially Fig. 1
Authors response:
Thank you. We have revised Figure 1 to improve its clarity.

Comment 5: Lack of comparison to non-LLM baselines or clinical risk scores, should be included
Authors response:
Thank you. We have added one extra subsection (4.6 Comparison with non-LLM techniques) to the results, which includes the performance of popular non-LLM models. Table 7 has also been added to show the performance of non-LLM techniques in different experimental settings.

Comment 6: No ablation studies to isolate impact of different components, should be included
Authors response:
Thank you. We have explored several settings during the design of the framework, and we selected the best settings to investigate different modalities, fusion mechanisms, and ensemble learning. Note that the main phases of the proposed framework (e.g., tokenization, padding, fine-tuning, etc.) are standard and mandatory components of NLP pipelines.

Comment 7: Lack of complexity analysis of Fine-tuning LLMs, should be included
Authors response:
Thank you for your feedback. While we appreciate your suggestion to include a complexity analysis of fine-tuning LLMs, our study focuses primarily on improving the performance of mortality prediction using a novel ensemble framework of multimodal, unstructured data. Including such an analysis would shift the scope away from the paper's main objectives. We believe the current content provides sufficient insight for the intended audience, and we will leave this analysis for future work, as we pointed out in Section 5 (Limitations and Future Directions).

Comment 8: Limited exploration of early fusion or other fusion strategies, should be included
Authors response:
Thank you. We have added one extra subsection (4.4 Results based on multimodal data: early fusion) to the results to explore the performance of the best-performing LLM/LM models for data of discharge summaries and radiology reports.

Comment 9: Missing statistical significance tests for model comparisons
Authors response:
Statistical significance analysis of the evaluated models is done using the ANOVA technique, and we reported these details in Section 4 (Results and Discussion).

Comment 10: Inadequate discussion of limitations and potential biases

Authors response:

We have dedicated Section 5 to discuss the limitations, biases, and future directions.

Reviewer 2

Comment 1: The paper would benefit from condensing the points of focus into specific Research Questions (RQ), which can then be referred to in the discussions later in the paper to connect the relevant findings with specific RQs for a more complete discussion of the results.
Authors response:

Thank you. We have added the research questions to Section 1 (Introduction) and updated Section 4 (Results and Discussion) to answer them comprehensively.

Comment 2: F1 score is the metric for evaluation used in this paper, which might be a common method in this use case but the authors still needed to substantiate with appropriate citations for their reasoning to use it.
Authors response:
We have used various metrics including accuracy, precision, recall, and F1-score. The values of all of these metrics are consistent, so we used the F1-score in the comparison. To support our methodology of comparison, we added extra citations for papers that follow a similar way of using the F1-score in the comparison.

Comment 3: The paragraphs need to have a 'justified' alignment.

Authors response:
Done.

Comment 4: The tables need to be more legible.

Authors response:
Done

Comment 5: The results could be better presented with some form of visualization.

Authors response:
We have added one Figure (Figure 3) in Section 4.5 that summarizes the overall performance of the evaluated models in the study, highlighting the best-performing techniques for mortality prediction.

Reviewer 3

Comment 1: It is not clear how the patients were assigned to "mental disorder". The authors mention ICD-10 code beginning with "F" relating to mental health disorders. However, it is not clear how the "F" ICD-10 code is used. Is a single "F" code in the patient's entire history sufficient to set this flag in the input feature? Is the feature binary (has/has not mental health disorder)? Is the specific ICD-10 code included as a feature.

Authors response:
Thank you.
Note that the morbidities of the ICU patients are coded using the ICD-10 international standard. We used this standard to collect the list of patients that have mental disorders, which has the following codes in ICD-10. F00-F09 (organic, including symptomatic, mental disorders), F10-F19 (mental and behavioral disorders due to psychoactive substance use), F20-F29 (schizophrenia, schizotypal, and delusional disorders), F30-F39 (mood [affective] disorders), F40-F48 (neurotic, stress-related, and somatoform disorders), F50-F59 (behavioral syndromes associated with physiological disturbances and physical factors), F60-F69 (disorders of adult personality and behavior), F70-F79 (intellectual disabilities), F80-F89 (pervasive and specific developmental disorders), F90-F98 (behavioral and emotional disorders with onset usually occurring in childhood and adolescence), and F99 (unspecified mental disorder). For more details, please access the following link https://icd.who.int/browse10/2019/en#/V

Comment 2: The authors did not make clear the patient distribution in MIMIC IV such as how many of the patients suffered from mental disorders and how many did not, or how many died in ICU and how many **survived** (for at least one year). This is particularly important in choosing the data - were all patients who died in ICU included and a random selection of the remaining patients included to balance the study dataset then chosen? The study could have been made more robust by choosing multiple partitions of died/not died patients.

Authors response:
Since this study is about mortality prediction of mental disorders patients, all subjects of the study had a mental disorder. For the design of the evaluation, we have filtered out the patients with mental disorders, and the total number is 29,419. These patients include patients who died in the ICU in-hospital (4,684 subjects) or died after discharge (1,273 subjects), with a total of subjects with mental disorders of 5,957 subjects. To balance the prediction task, we randomly sampled 5,957 subjects out of the 29,419 with the alive condition. This means we had 11,914 subjects (alive/dead) with discharge summary reports.
Since our study also combined discharge summaries with radiology reports, we found that some of the subjects did not have radiology reports, and thus reduced the number of subjects with discharge summaries and radiology reports to 10,243 (5,121 dead).

We have clarified this in the description of the study dataset by editing Section 3.1.

Comment 3: I would like to have seen a more detailed description of the dataset used for the study. As far as I can tell, the input consists of Discharge Summary, Radiology, a flag for mental disorder and a target variable (died in ICU).
Authors response:

Based on the previous comment, we have edited the dataset Section (Section 3.1) with more details about the dataset and the selection criteria.

Comment 4: Description of the training/validation/testing regime is limited. The authors mention the dataset was partitioned 90% training/validation and 10% testing. However, there is no description of the number or repetitions of the experiments, whether different partitioning was used in different experiment runs, etc.

Authors response:
Thank you. In this study, we mainly explore the capabilities of the medically pretrained LM/LLM models. These models have already been trained, optimized, and fine-tuned to huge amounts of medical data. As a result, the models' weights have the medical knowledge to converge to the optimal performance for the tested task quickly. In our experiments, we did fine-tuning using two optimization techniques LoRA and quantization. At the same time, our dataset is sufficient enough to fine-tune the selected models. We tested our models with different random splits of data, and we found no significant difference in the results.
On the other hand, the classical deep learning models with random initial weights have been validated using the 5-fold cross validation and the average results have been reported.

Comment 5: The results reported are the "best" for each model and experiment. It would be useful to know the range of results and whether the "best" is significantly different from the usual or the worst result.

Authors response:
Statistical significance analysis of the evaluated models is done using the ANOVA technique, and we reported these details in Section 4 (Results and Discussion).

Comment 6: The paper addresses the question of how various LM/LLM models compare in predicting death in ICU. There are already clinical multi-morbidity models in use that generate mortality risk predictions (over various time horizons - 90 day, 1 year, 5 year). These models are already accepted and in clinical use. I think the paper would be strengthened by comparing with one or more of these models.

Authors response:
Thank you. To the best of our knowledge, there are no medically accepted calculators for mortality prediction in the literature, especially using deep learning models. The existing calculators, such as SAPS, APACHE, MPM, and SOFA tools, are not multimodal and are based on limited numerical features such as physiological variables, vital signs, urinary outputs, nerve scores, age, and other conditions. In this study, we mainly concentrated on the role of unstructured clinical notes in predicting the mortality of mentally

disordered patients. To the best of our knowledge, no study in the literature has explored the capabilities of LM/LLM to understand text data to predict mortality in ICU for mental disorder patients.

Comment 7: The authors have relied heavily on pre-prints or unpublished archive references (12 out of 67 references). I think this is not a good idea.
Authors response:
Thank you. We have reduced the preprints in the references list to only 5 highly cited preprints. These cited preprints introduce some medically pre-trained models that we review in the study.

Comment 8: To make it easier for the reader to pick out the best performing models, the table rows containing these should be bolded/highlighted.
Authors response:

We highlighted in bold the best performing models in all tables. We have also added a summary Figure (See Figure 3) that highlights the best-performing techniques.

Comment 9: As far as I can see, the datsets used for training are not made available to the reader.
Authors response:
Based on the data agreement we signed with MIMIC, we are not allowed to share the data. We have added a data availability statement after the conclusion Section with a link to MIMIC dataset.

Comment 10:
Corrections
Introduction
anorexia Nervosa -> anorexia nervosa
Section 3.2
(1st sentence) particularly those that involves -> particularly those that involve

Section 3.3
Figure 1 illustr2ates -> illustrates

Authors response:

All the raised typos and grammar issues have been resolved. Following the editing comments received from the English editing service, we have thoroughly revised the language of the manuscript.

Comment 11:
References
There are many malformed (missing key information such as publisher, etc). These include

There are many malformed references for URLs (author attribution is incorrect). These include [2], [3], [4], [5], [6]

Some references use full journal name, others abbreviations. Referencing should be consistent - suggest using teh full journal name throughout - see [19], [56], [58]

Authors response:
We have checked all the references and fixed all the raised issues with the reference details.

Comment 12: There is inconsistent capitalisation of journal names. Some capitalise each word, others only the first word. Again, the authors are advised to be consistent and to adopt the standard of the journal to which the article has been submitted.

Authors response:
We unified the format of the journal name in all references.

Comment 13: Similarly, there is inconsistent capitalisation in article titles. Some Some capitalise each word, others only the first word. In some, the first word after a colon is capitalised, while in others it is not. Also, there are instances where capitals should be used to refer to an artefact - e.g MIMIC IV rather than Mimic iv (ref [54]) and Mental-LLM rather than Mental llm (ref [26]). Again, the authors are advised to be consistent and to adopt the standard of the journal to which the article has been submitted.
Authors response:
We resolved the raised issue with the reference titles and checked the consistency of all references.
