# Supplementary material for: Mortality prediction for ICU patients with mental disorders using large language models ensemble and unstructured medical notes
Source: PLoS One. 2025 Sep 19;20(9):e0332134. doi: 10.1371/journal.pone.0332134 (PMC12448993; doi:10.1371/journal.pone.0332134)
Supplement: Attachment — Submitted filename: Response file 2nd round.docx [file pone.0332134.s002.docx]

Response to the Reviewers on the manuscript entitled: **Mortality Prediction for ICU Patients with Mental Disorders Using Large Language Models Ensemble and Unstructured Medical Notes**

Waleed Nazih, Tamer Abuhmed, Meshal Alharbi, Shaker El-Sappagh

Manuscript ID: PONE-D-24-32699R2

We thank the reviewers and editor for their valuable comments that significantly helped us to enrich the quality of the manuscript. We've carefully considered the reviewers' comments in our revision. We have tried our best to incorporate the reviewers' and editors' suggestions into the revised version of the manuscript.

To address each reviewer's comments carefully, we would like to respond to them point by point. All changes have been highlighted in the manuscript using the red color.

Best regards,

Authors

**Editor comment**

Thank you for submitting your manuscript to PLOS ONE. After careful consideration, we feel that it has merit but does not fully meet PLOS ONE's publication criteria as it currently stands. Therefore, we invite you to submit a revised version of the manuscript that addresses the points raised during the review process.

**Authors’ response**

Thank you for your feedback and the minor revision decision.

**Reviewer 3**

**Reviewer comment:**

I thank the authors for addressing my comments. I am happy with the responses for all but three of my comments. I commend the authors on the modifications they have made to the paper. It is indeed improved and will be a significant contribution.

**Authors’ response**
Thank you so much for your encouraging and positive feedback.

**Reviewer comment:**

Firstly, I am still unsure about the inclusion criteria for patients. The authors clearly state the set of ICD codes that identify mental disorders. What I am missing is if inclusion was based on the presence of one or more of these codes in the latest admission, or whether the inclusion is based on the occurrence of one of these codes in the entire patient history. For instance, if a patient has more than one admittance in the database and an early admittance includes an F50-F59 code, but the latest admittance does not show this code, would the patient be included in the study?

**Authors’ response**

Thank you for your valuable feedback. We agree that the initial description of the inclusion criteria requires additional clarification. To address your concern, this study does not consider the complete admission history of patients or multiple admissions. Instead, the inclusion criteria were based on the presence of mental disorder ICD-10 codes in the patient’s diagnosis during any of their admissions. For such cases, we included the discharge and radiology reports of the specific admission where these codes were recorded. This approach ensures that the analysis focuses solely on the textual data directly associated with admissions where mental disorders were explicitly documented. We revised Section 3.1 to explicitly clarify this point.

**Reviewer comment:**

Also, is the mental disorder required to be the primary diagnosis or just included as one of the diagnosis codes? In my experience, hospital records may include up to 20 diagnosis codes.

**Authors’ response**
Thank you.

It is well-known that hospital records may include up to 20 diagnosis codes. In our study, we focused mainly on the doctors’ reports of patients with mental disorders. Note that the collection of these mentally disordered patients was filtered out based on other MIMIC-IV tables and the standard mental disorders ICD codes. However, our LLM models were fine-tuned only on the text reports. In MIMIC-IV, it is not possible to know which is the primary diagnosis of the patient, as the doctors include several diagnoses during the patient's admissions. As a result, we did the following: (1) identify the patients that have mental disorders in their list of diagnoses, (2) filter out the mortality status of these patients.

As hospital records can contain multiple diagnosis codes, our inclusion criteria aimed to capture all admissions where mental disorders were identified, ensuring that the analysis comprehensively reflects the potential impact of these conditions on patient outcomes, irrespective of their ranking in the diagnosis list.
We revised Section 3.1 to explicitly clarify this point.

**Reviewer comment:**

Next, I am still unsure about the structure feature vectors. My understanding is that the only attributes presented include either/both discharge/radiology report and a target, with no other demographic or clinical history information being included. I think this needs clarifying.

**Authors’ response**
Thank you.

Yes. Your understanding is correct, and we depend only on the text data of discharge and radiology reports. We clarify the point in the revised manuscript in “Section 3.3 Proposed Framework.”

**Reviewer comment:**

Lastly, I feel that not taking into account comorbidities may introduce some bias into the data. What fraction of the patient cohort had potentially life threatening comorbidities (as well as mental disorder)? That is, are mental disorders independent of other conditions? Were comorbidities equally distributed across the [died |did not die] partitions? I understand that this is not a clinical paper, it is about experimenting with the capabilities of medically trained LLM/LM models. Nevertheless, it is important to understand and acknowledge the characteristics of the data as they impact (or do not impact) on prediction.

**Authors’ response**
Thank you.

As you mentioned, this is not a medical study. We focused mainly on testing LLM models on text data only of the reports without considering any structured data, including the patient’s comorbidities, to understand the narrative reports of patients and predict mortality in the ICU.

Although our models are trained solely on text data without including any structured data, our used dataset is balanced regarding the existence of comorbidities. In other words, the percentages of alive and dead patients are balanced for every severe comorbidity, including Congestive Heart Failure, Cerebrovascular Disease, Dementia, Chronic Pulmonary Disease, Rheumatic Disease, Peptic Ulcer Disease, Severe Liver Disease, Chronic Diabetes Renal Disease, among others. This means that the patient’s comorbidities are neutral regarding the alive and dead status of the patient. Based on your comment, this note has been highlighted in “Section 3.1: Dataset.”

However, considering structure data, including patient comorbidities, will be investigated deeply in our future study. The list of limitations and future research directions in the revised manuscript (i.e., Section 5) has been updated to highlight this point.

**Reviewer comment:**

Spelling mistake: Section 4.4, 1st paragraph - archives -> achieves

**Authors’ response**
Thank you.

Fixed.

**Reviewer 4**

**Reviewer comment:**

The article has successfully met all the necessary requirements for publication, adhering to the stipulated guidelines and standards. After thoroughly reviewing its content, structure, and alignment with the publication's objectives, I am pleased to confirm that there are no outstanding issues or points of concern. As such, I have no further questions or comments to raise at this stage.

**Authors’ response**
Thank you for accepting our revised manuscript.
