# Supplementary material for: Mortality prediction for ICU patients with mental disorders using large language models ensemble and unstructured medical notes
Source: PLoS One. 2025 Sep 19;20(9):e0332134. doi: 10.1371/journal.pone.0332134 (PMC12448993; doi:10.1371/journal.pone.0332134)
Supplement: Attachment — Submitted filename: LLM paper R4 responses.docx [file pone.0332134.s003.docx]

# Response to the Reviewers on the manuscript entitled: **Mortality Prediction for ICU Patients with Mental Disorders Using Large Language Models Ensemble and Unstructured Medical Notes**

Waleed Nazih, Tamer Abuhmed, Meshal Alharbi, Shaker El-Sappagh

Manuscript ID: PONE-D-24-32699R3

We thank the reviewers and editor for their valuable comments that significantly helped us to enrich the quality of the manuscript. We've carefully considered the reviewers' comments in our revision. We have tried our best to incorporate the reviewers' and editors' suggestions into the revised version of the manuscript.

To address each reviewer's comments carefully, we would like to respond to them point by point. All changes have been highlighted in the manuscript using the red color.

Best regards,

Authors

Reviewer #5:

**Reviewer comment:**

Paper is well-written, clear and easy-to-follow. The technical soundness and novelties are strong. The experiments are extensive that show all aspects to assess the effectiveness of methods. I believe this work is sufficient to be published.

**Authors’ response:**

Thank you so much for accepting the manuscript.

**Reviewer comment:**

Fig 2 quality should be increased.

**Authors’ response:**

The quality of the figure has been enhanced (Figure 4 in the revised version).

Reviewer #6:

**Reviewer comment:**

The paper doesn’t compare the models to basic baselines like logistic regression or ICU scoring systems (e.g., SOFA, SAPS), which makes it hard to judge if these LLMs are actually better.

**Authors’ response:**

Thank you. Section “4.6 Comparison with non-LLM techniques” discusses the results of non-LLM models. In addition, Table 7 reports the results of these models. We focused on the advanced techniques, including CNN and LSTM models.

**Reviewer comment:**

They use majority voting for the ensemble, but don’t explain why that method was picked or if other fusion methods were tested.

**Authors’ response:**

Thank you. We have clarified our rationale for choosing majority voting in the revised manuscript. Majority voting is a widely used and effective ensemble technique due to its simplicity, interpretability, and robustness, especially when the individual models have comparable performance and are diverse in nature. Given these properties, and to maintain a focused experimental scope, we selected majority voting as a representative and interpretable fusion method. We did not explore other fusion strategies in this study, as our primary objective was to assess the viability of the proposed models within a practical ensemble framework. We justify our selection of this method in the Proposed Framework Section (Section 3.3).

**Reviewer comment:**

It’s unclear how long clinical notes (which can be huge) were handled. Were they cut off? Summarized? This could affect the results a lot.

**Authors’ response:**

Thank you. We appreciate the reviewer’s attention to this important aspect. We have now clarified in Section 3.1 (Dataset) how long clinical notes were handled. Specifically, we applied a maximum token limit consistent with the input capacity of the underlying language model, truncating any notes that exceeded this threshold from the end. This approach is commonly used in clinical NLP settings where models have fixed input sizes.

**Reviewer comment:**

Only one dataset (MIMIC-IV) is used, and it’s just for patients with mental disorders. It’s hard to know if the results would work for other types of patients or in different hospitals.

**Authors’ response:**

Thank you. Our study focuses specifically on ICU patients with mental disorders, a population that has been historically underrepresented in predictive modeling research. The choice of MIMIC-IV was driven by its rich, publicly available clinical notes and the ability to identify a well-defined cohort with mental health diagnoses. While this does limit direct generalization to other patient populations or hospital settings, our methodology, particularly the use of diverse LLMs/LMs and decision fusion techniques, is designed to be broadly applicable. We have now added a discussion of this limitation and the potential for future validation on external datasets in the future work (Section 5).

**Reviewer comment:**

The results don’t include confidence intervals or show if the improvements are statistically solid. Some scores are very close — we don’t know if the gains are real.

**Authors’ response:**

Thank you. We would like to clarify that we conducted statistical testing to assess the robustness of the performance differences observed. Specifically, we employed ANOVA (Analysis of Variance) tests with a significance level of α = 0.05 to evaluate the statistical significance of model performance across different configurations. This is now explicitly stated and emphasized in Section 4 of the revised manuscript. Additionally, while confidence intervals were not originally included in the reported metrics, we acknowledge their value in interpreting close performance differences and plan to incorporate confidence interval reporting in future work.

**Reviewer comment:**

The paper doesn’t explain what the models are learning or how decisions are made — there’s no interpretability, which is important in clinical work.

**Authors’ response:**

Thank you. We fully agree that interpretability is critical in clinical settings. Our current focus is to establish the feasibility and effectiveness of using LLMs and ensemble methods for ICU mortality prediction in patients with mental disorders. While this work primarily emphasizes predictive performance, we recognize that understanding model decision-making is essential for clinical adoption. We have now included a dedicated discussion in the Limitations section (Section 5) acknowledging the absence of interpretability techniques (e.g., attention heatmaps, SHAP, or saliency-based methods) and highlighting the need for future work to incorporate model explanation mechanisms that can provide clinicians with transparent, actionable insights.

**Reviewer comment:**

Writing is a bit wordy in places and technical details (like preprocessing or how inputs were built) could be clearer.

**Authors’ response:**

Thank you. We have carefully revised the manuscript to improve clarity and conciseness. We have streamlined the writing in Sections 3.1 and 3.3 to reduce redundancy and enhance readability. Additionally, we have clarified the preprocessing steps and how input sequences were constructed for each model, including tokenization, padding, and handling of sequence lengths. These changes aim to ensure the technical pipeline is more transparent to readers. We believe the revised text improves both accessibility and precision.

Reviewer #7:

**Reviewer comment:**

Overall: This manuscript presents a novel approach to predicting ICU mortality in patients with mental disorders using large language models and unstructured clinical notes, demonstrating considerable contributions with impressive performance results.

Strength:
- The introduction provides a comprehensive background on mental health disorders with current statistics from reputable sources (WHO, NIH). It also thoroughly reviews existing approaches for ICU mortality prediction. Based on these bases, this study effectively establishes the novelty of using LLMs for mortality prediction in mentally disordered ICU patients.

- Research questions (RQ1-RQ7) are specific and directly address identified gaps in the literature. The contributions are clearly articulated and aligned with the research objectives.

- The dataset description is presented well, providing clear information about the MIMIC-IV clinical notes and inclusion criteria for mental disorder patients.

- Although the methodology section lacks some foundations to make it more rigorous, it is generally good and can be considered one of the most important contributions of the paper due to its novelty.

- The experimental setup is fair, and the experimental results are impressive.

**Authors’ response:**

Thank you for your positive feedback.

**Reviewer comment:**

Although there are several grammatical and typographical errors, they are not significant. However, there are some parts in both methods and experiments that need to be revised.

**Authors’ response:**

We thank the reviewer for pointing this out. We have thoroughly revised the manuscript to correct grammatical and typographical issues and to improve clarity and flow throughout.

**Reviewer comment:**

- The introduction is excessively detailed in certain areas, along with problems in paragraph division (particularly in paragraphs 3 & 4, lines 67-101), creating a disjointed narrative. As a results, research gaps are scattered throughout rather than cohesively presented.

**Authors’ response:**

Thank you. In the revised manuscript, we have restructured the *Introduction* to improve coherence and narrative flow. Specifically, we have condensed overly detailed sections and reorganized mentioned paragraphs to improve paragraph unity and logical progression. We also revised the presentation of research gaps to ensure they are clearly and cohesively articulated toward the end of the introduction. These revisions aim to provide a more focused and readable motivation for our study while emphasizing the novelty and significance of our contributions.

**Reviewer comment:**

- Despite the adequate general motivation, it is not as compelling as it could be due to the fragmented presentation of research gaps. Another reason is that the connection between mental disorders and ICU mortality risk could be more explicitly linked to the proposed technical approach.

**Authors’ response:**

Thank you.

We have improved the structure and clarity of the *Introduction* Section by consolidating and reorganizing the discussion of research gaps to present a more cohesive and compelling narrative. Additionally, we have strengthened the linkage between the clinical context, i.e., the elevated mortality risk associated with mental disorders in ICU settings, and our proposed technical approach using language model ensembles on unstructured clinical notes. These changes aim to more clearly convey why this patient population requires specialized modeling and how our approach is designed to address those unique challenges. We believe this improves the overall motivation and coherence of the study.

**Reviewer comment:**

- There are many research questions (RQ1-RQ7). It would be better if these questions can be clustered into logical categories (e.g., "Model Architecture Questions," "Data Modality Questions").

**Authors’ response:**

Thank you. We agree that clustering the research questions into thematic groups improves clarity and helps guide the reader through the objectives of the study. In the revised manuscript, we have reorganized RQ1–RQ7 under three logical categories: Model Architecture and Optimization (e.g., RQ1, RQ3, RQ4, RQ7), Data Modality and Representation (e.g., RQ2, RQ6), and Applicability of Unstructured Clinical Text (e.g., RQ5).

This reorganization is now reflected in the *Introduction Section* to improve readability and highlight the methodological structure of the work.

**Reviewer comment:**

The statement "The ensemble of heterogeneous language models (LMs) and LLMs effectively addresses these limitations" in the abstract is imprecise, as LLMs are inherently a subset of language models.

**Authors’ response:**

Thank you.

We agree that LLMs are a subset of language models and that the phrasing in the original abstract may cause confusion. In the revised version, we have updated the statement to: *“The ensemble of heterogeneous language models, including both conventional LMs and large language models (LLMs), effectively addresses these limitations.”* This change aims to clarify the intended contrast between models of different scales and architectural capabilities within the broader category of language models.

**Reviewer comment:**

The Methods section lacks a sufficient explanation for using Low-Rank Adaptation (LoRA) over alternative fine-tuning approaches. Figure 2 appears to be directly reused from the original LoRA paper rather than being redrawn specifically for this study's context.

**Authors’ response:**

Thank you.

We have revised the *Methods* section to provide a clearer rationale for selecting Low-Rank Adaptation (LoRA) over other fine-tuning techniques. Specifically, LoRA was chosen due to its efficiency in reducing the number of trainable parameters, its compatibility with large models in resource-constrained environments, and its strong performance in prior medical NLP studies. This choice allowed us to fine-tune a diverse set of LLMs and LMs with minimal computational overhead while maintaining high performance. Regarding Figure 2 (Figure 4 in the revised manuscript), we acknowledge that it closely resembles the original figure from the LoRA paper. In the revised manuscript, we have replaced it with a custom redrawn version tailored to our specific model setup and training context, and we have properly cited the original source to avoid any issues with reuse.

**Reviewer comment:**

- Data preprocessing steps are minimally described, with limited details on tokenization and handling of medical terminology.

**Authors’ response:**

Thank you. We have expanded the *Methods* section to provide a more detailed description of the data preprocessing pipeline. This includes a clearer explanation of the tokenization process, which was performed using model-specific tokenizers compatible with each LLM and LM architecture (e.g., WordPiece for BERT-based models, SentencePiece for LLaMA-based models). We also clarify that clinical notes were minimally preprocessed to preserve domain-specific medical terminology, which is critical for effective language model performance in the clinical context. Specifically, we retained all medically relevant terms and abbreviations, removed only redacted identifiers and non-informative characters (e.g., excessive punctuation, repeated whitespace), and avoided aggressive normalization steps such as spelling correction or term standardization to prevent loss of semantic fidelity. These details have now been added to Section 3.3.

**Reviewer comment:**

The claim that "the utilized dataset is balanced with respect to the presence of severe comorbidities" (lines 328-332) lacks supporting evidence such as statistical tests or distribution visualizations.

**Authors’ response:**

Thank you. We acknowledge that the original statement lacked empirical support. To address this, we have revised the manuscript to clarify that while the dataset was balanced with respect to the mortality outcome (i.e., an equal number of deceased and surviving patients with mental disorders), no explicit stratification or matching was performed based on comorbidity severity. We have now removed the unsupported claim about comorbidity balance and added a note in the *Limitations* section to acknowledge this point.

**Reviewer comment:**

- Several instances of redundant terminology appear throughout the manuscript (e.g., "LLM models" where"LLM" already means "Large Language Model"), and Section 3.2 contains a duplicated colon at the end of paragraph 2 (line 368).
**Authors’ response:**

Thank you. We have fixed this issue in the revised manuscript.

**Reviewer comment:**

Table 2 is too small, making it difficult to verify its contents.

**Authors’ response:**

Thank you. We have fixed this issue in the revised manuscript.

**Reviewer comment:**

Some sentences have awkward phrasing or grammatical issues (e.g., lines 512-513, 601, 652-654).

**Authors’ response:**

Thank you. We have rephrased the sentences and fixed grammatical issues in the revised manuscript.

**Reviewer comment:**

The manuscript lacks visualizations of data distribution and experiments such as mental disorder categories, length distributions of clinical notes, model performance across different patient subgroups, or confusion matrices for the best-performing models.

**Authors’ response:**

Thank you.

Figure 1 has been added to show a histogram showing the distribution of clinical note lengths (in words) across the dataset. Figure 2 has been added to show a bar chart summarizing the frequency of major mental disorder categories (e.g., schizophrenia, depression, bipolar disorder) based on ICD-10 codes. Figure 6 has been added to show the confusion matrix for the best-performing model to highlight the distribution of true positives, false positives, true negatives, and false negatives for the best performing models. Figure 2 of the LoRA technique has been refined.

**Reviewer comment:**

- The preprocessing for datasets is not described to ensure reproducibility and justification.

**Authors’ response:**

Thank you. We have expanded the *Methods* section to provide a more detailed description of the data preprocessing pipeline. This includes a clearer explanation of the tokenization process, which was performed using model-specific tokenizers compatible with each LLM and LM architecture (e.g., WordPiece for BERT-based models, SentencePiece for LLaMA-based models). We also clarify that clinical notes were minimally preprocessed to preserve domain-specific medical terminology, which is critical for effective language model performance in the clinical context. Specifically, we retained all medically relevant terms and abbreviations, removed only redacted identifiers and non-informative characters (e.g., excessive punctuation, repeated whitespace), and avoided aggressive normalization steps such as spelling correction or term standardization to prevent loss of semantic fidelity. These details have now been added to Section 3.3.

**Reviewer comment:**

- Is the 1B parameter threshold for distinguishing between LMs and LLMs arbitrary or based on evidence?
**Authors’ response:**

Thank you.

We acknowledge that the 1 billion parameter threshold used to distinguish between LMs and LLMs is somewhat heuristic and not formally standardized in literature. However, this threshold is commonly referenced in contemporary LLM research and reflects a practical distinction based on observed shifts in model behavior, capabilities, and resource requirements. Specifically, models with over 1B parameters typically demonstrate enhanced generalization, emergent reasoning abilities, and higher computational demands, characteristics broadly associated with LLMs. This operational definition has been adopted in several studies (e.g., [1], [2]) and aligns with how model families such as GPT, PaLM, and LLaMA are categorized. We have clarified this rationale in the revised manuscript (Section 3.2) to avoid ambiguity and acknowledge the evolving nature of these definitions in the field.

[1] Brown, T., Mann, B., Ryder, N., Subbiah, M., Kaplan, J. D., Dhariwal, P., ... & Amodei, D. (2020). Language models are few-shot learners. *Advances in neural information processing systems*, *33*, 1877-1901.

[2] Touvron, H., Lavril, T., Izacard, G., Martinet, X., Lachaux, M. A., Lacroix, T., ... & Lample, G. (2023). Llama: Open and efficient foundation language models. *arXiv preprint arXiv:2302.13971*.

**Reviewer comment:**

- Currently, the number of datasets which are employed is not enough, making it difficult to confirm the robustness and generalizability of the proposed method's effectiveness. There is a need to consider expanding the experimental scope in terms of data.

**Authors’ response:**

Thank you. As noted, our current study focuses exclusively on the MIMIC-IV dataset, which offers a rich and well-structured corpus of ICU clinical notes and enables a targeted investigation of mortality prediction in patients with mental disorders, a population that is underrepresented in existing literature. However, we acknowledge that relying on a single dataset limits the broader generalizability of our conclusions. To address this, we have added a note in the *Limitations* section (Section 5) emphasizing the need for future work to validate the proposed framework on external datasets from other institutions or populations (e.g., eICU, HiRID, or institutional EHRs). These expansions will be crucial to assess the model’s robustness across different clinical environments and documentation practices. We consider our work a first step toward that broader goal.
