# Supplementary material for: Mortality prediction for ICU patients with mental disorders using large language models ensemble and unstructured medical notes
Source: PLoS One. 2025 Sep 19;20(9):e0332134. doi: 10.1371/journal.pone.0332134 (PMC12448993; doi:10.1371/journal.pone.0332134)
Supplement: Attachment — Submitted filename: LLM paper R5 responses.docx [file pone.0332134.s004.docx]

# Response to the Reviewers on the manuscript entitled: **Mortality Prediction for ICU Patients with Mental Disorders Using Large Language Models Ensemble and Unstructured Medical Notes**

Waleed Nazih, Tamer Abuhmed, Meshal Alharbi, Shaker El-Sappagh

Manuscript ID: PONE-D-24-32699R5

We thank the reviewers and editor for their valuable help in enriching the quality of the manuscript. Since all reviewers have accepted the manuscript and confirmed that authors have addressed the raised comments, we have tried our best to proofread the final manuscript and ensure that it meets the standards of written academic English. We also confirmed that the manuscript has been screened for unintentional plagiarism and that all sources are properly cited. We have also submitted the manuscript to the similarity evaluation service “Turnitin” and attached the report to this submission.

The authors attached the reviewers' confirmations below.

Best regards,

Authors

**Reviewer #5:** Latest revision is well-written. Idea is clear. Experimental quantitative results are sufficient. I believe this work is ready for publication.

**Authors' response:** Thank you for accepting the manuscript.

**Reviewer #8:** All comments have been addressed. The paper can be accepted for publication.

**Authors' response:** Thank you for accepting the manuscript.

**Reviewer #9:** I appreciate the authors’ thoughtful and well-structured responses to the initial comments. Most concerns have been addressed with appropriate revisions and clarifications.

**Authors' response:** Thank you for accepting the manuscript.

**Reviewer #10:** Authors have addressed all the raised concerns by the reviewers. The quality of the paper is now in a standard scale and ready to be published.

**Authors' response:** Thank you for accepting the manuscript.

**Reviewer #11:** In summary, the authors have rigorously addressed previous reviewer concerns and the work represents the solid scientific contribution with clear clinical relevance. They had conducted extensive ablation studies and justified theories to demonstrate their hypotheses. Therefore, I recommend an acceptance for this manuscript.

**Authors' response:** Thank you for accepting the manuscript.
